# Gastrointestinal protozoan and helminth parasite infections in captive chameleons in Germany

**DOI:** 10.1007/s00436-026-08704-3

**Published:** 2026-06-08

**Authors:** Paula Sapion-Miranda, Anja Taubert, Carlos Hermosilla, Malek J. Hallinger

**Affiliations:** 1 Institute of Parasitology, Justus Liebig University Giessen, Giessen, Germany; 2 exomed GmbH, Marburg, Germany

**Keywords:** Chameleons, Parasites, Exotic pets, Reptile medicine, Wildlife

## Abstract

Within lizards, chameleons (family Chamaeleonidae) represent a distinctive and highly specialized clade of insectivorous reptiles. Chameleons have become more popular as reptile pets in recent years worldwide, due to their feasible husbandry and attractiveness. To date, not only reptile pet numbers but also health-compromising endoparasite infections are rising. In the current study, we analyzed a total of 670 faecal samples of 25 different chameleon species kept as pet animals all over Germany. In total, 223 animals (33%) were found to harbor gastrointestinal parasites. In total, 10 different parasite taxa were diagnosed in chameleon faecal samples. The most prevalent parasite stages were oxyurid eggs (14.1%), followed by coccidia oocysts (7.8%) (e.g. *Isospora jaracimrmani*, *Isospora* spp., *Choleoeimeria* spp., *Eimeria* spp.), flagellated protozoan trophozoites (6.3%) (e.g. *Leptomonas* spp., *Tritrichomonas* spp., *Proteromonas* spp.), heterakid eggs (3.6%), thin-shelled nematode eggs (3.6%), digenean trematode eggs (0.6%), pentastomid eggs (0.3%), ascarid eggs (0.2%), *Entamoeba* spp. cysts (0.2%) and physalopteroid eggs (0.2%). Among current chameleon species, veiled chameleons (*Chamaleo calyptratus*) showed a significantly higher prevalence of oxyurid infections (64%) than other chameleon species. Co-infections were diagnosed in 9.8% (22/223) of faecal samples. When performing necropsies, 19/31 chameleons showed parasite-induced pathologies based on histopathological examinations, including one animal being infected with 4 parasite species (*Leptomonas* spp., *Tritrichomonas* spp., *Foleyella furcata* and *Hexametra angusticaecoides)*, thereby most likely impacting animal’s welfare.

**Supplementary information:**

The online version contains supplementary material available at 10.1007/s00436-026-08704-3.

## Introduction

To date, reptile husbandry is increasing in popularity since captive-bred reptiles can easily be obtained as exotic pets (Carpenter et al. 2004). However, not all reptiles are available from breeders, and enthusiasts seeking exotic options may turn to wild-caught specimen with potentially unknown origin (Hallinger et al. 2018; Marshall et al. 2020; Toland et al. 2020). This ambiguity contributes to the introduction of numerous potentially invasive (neozoan) pathogens of these reptiles, including both ecto- and endoparasites (Rataj et al. 2011).

Both wild and captive reptiles are frequently infected with endoparasites, such as apicomplexans, flagellated protozoa, nematodes, cestodes, trematodes, and pentastomids (Frank 1985; Paré 2008; Pasmans et al. 2008; Pantchev 2013; Hallinger et al. 2018). While many of these parasites typically have low pathogenicity in their original habitats, they can significantly impact the animal’s health if environmental conditions change (Wilson and Carpenter 1996). Hence, several risk factors like inadequate temperatures, stress, and high-density stocking affect the reptile’s immune system (Bower et al. 2019). In this context, enhanced stress during trapping and transport of wild-caught reptiles often drives an increase in morbidity and mortality rates (Bower et al. 2019; Stets 2019).

In captive reptiles, a notable prevalence of gastrointestinal parasitoses is common, especially referring to parasites with monoxenous lifecycles (Schneller and Pantchev 2008; Pantchev 2013; Hallinger et al. 2019). This phenomenon can be attributed to the high environmental tenacity of certain endoparasite stages (e.g. oocysts, cysts and eggs) and short prepatencies of related parasites (Pasmans et al. 2008; Cervone et al. 2016; Hallinger et al. 2018). Distinct intrinsic factors, such as age, gender, genus and species or extrinsic factors like poor hygiene, stress and nutrition can significantly contribute to the development of clinical diseases in reptiles (Pasmans et al. 2008; Pantchev 2013; Hallinger et al. 2018). Moreover, inadequate housing conditions create a perfect environment for high parasite burdens and frequent reinfections (Pasmans et al. 2008; Pantchev 2013; Wolf et al. 2014; Hallinger et al. 2019). Recent studies have suggested that feeding insects also represents a significant risk factor of endoparasite transmission, requiring further investigations (Gałęcki and Sokół 2019; Müller et al. 2019).

Within lizards, chameleons (family Chamaelonidae) represent a distinctive and highly specialized clade of insectivorous reptiles. Chameleons, renowned for their attractive appearance, have been successfully bred in captivity for over four decades, while their trading dates to the early 1800s (Carpenter et al. 2004; Diaz et al. 2015).

Similar to other captive reptiles, chameleons often show a high prevalence of endoparasites, a health-compromising condition that has raised considerable concern among herpetologists (Okulewicz et al. 2015; Rom et al. 2018). Despite this fact, there remains a gap in understanding on parasite transmission, parasite-driven pathogenesis and clinical manifestations (Nash et al. 2023). Moreover, many chameleon species, such as the Belalanda chameleon (*Furcifer belalanda*) from Madagascar or the Nguru spiny pygmy chameleon (*Rhampholeon acuminatus*) from Tanzania, fall into categories of critically endangered (4%) animals, others as endangered (21%), vulnerable (12.2%) or nearly threatened (18%), due to factors like habitat loss, illegal exotic pet trade and others (The IUCN Red List of Threatened Species 2019).

In Germany, there are only a few studies on parasites affecting chameleons. Consistently, Biallas (2013) conducted an extensive study on 212 chameleon faecal samples to investigate the prevalence and detrimental effects of endoparasites in captive chameleons, considering animal risk factors like origin, age and sex. Further, Pantchev (2011) examined 66 chameleon faecal samples and provided an overview on most common parasite infections and related clinical symptoms. Stets (2019) analyzed 646 faecal samples of Panther chameleons (*Furcifer pardalis*), thereby comparing endoparasite infections in wild-caught and captive-kept animals in the Ukraine. Additionally, some case reports on distinct parasitoses and related treatments in common captive chameleons (e.g. *F. pardalis*,* Chamaleo calyptratus*) were recorded (Damiani et al. 2006; Reitl et al. 2021; Heng et al. 2023). Some reports documented endoparasitoses of wild-caught chameleons (Dillehay et al. 1986; Lhermitte-Vallarino et al. 2008; Heckmann 2014; Pellett et al. 2014; Collicutt et al. 2017; Eckhardt et al. 2019) mostly focusing on parasite species with heteroxenous lifecycles. Occasionally, studies on reptile endoparasites also included individual findings on the most commonly kept chameleon species (*Chamaelo calyptratus*) (Wilson and Carpenter 1996; Rataj et al. 2011; Okulewicz et al. 2015; Rom et al. 2018; Satour and Dewir 2018) but lacking both large sample numbers and representative data.

In general, the identification of pathogenic endoparasite species, including anthropozoonotic ones, is crucial for adequate targeted parasitological treatments, thereby minimizing both chameleon morbidity/mortality and transmission to pet owners (Pantchev 2011; Mendoza-Roldan et al. 2020). Therefore, the current study aimed to provide novel insights into the occurrence of gastrointestinal endoparasites in captive-kept chameleons in Germany by investigating a substantial sample size (*n* = 670) but also involving 25 different chameleon species to ensure a representative analysis. In addition to faecal analyses, we performed histopathological examinations on 31 deceased chameleons, providing a new baseline dataset on the presence of endoparasites in these popular herpetological pet animals.

## Materials and methods

### Faecal samples

**Fig. 1:**
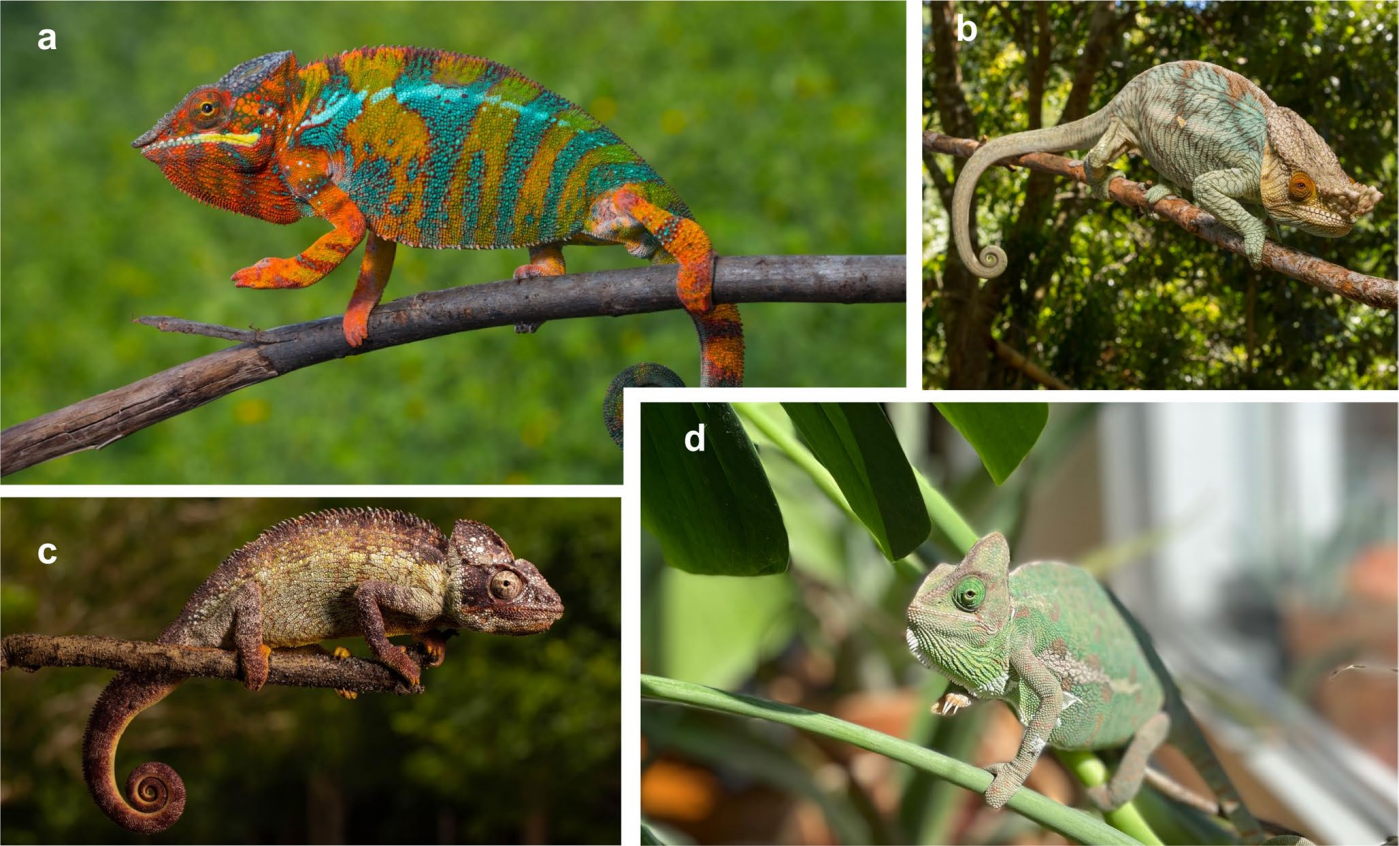
Exemplary images of different chameleon species investigated in this study (**a**) a male Panther chameleon (*Furcifer pardalis*), (**b**) a male Parson’s chameleon (*Calumma parsonii*), (**c**) a male Malagasy giant chameleon (*Furcifer oustaleti*), (**d**) a female Veiled chameleon (*Chamaelo calyptratus*).

**Fig. 2:**
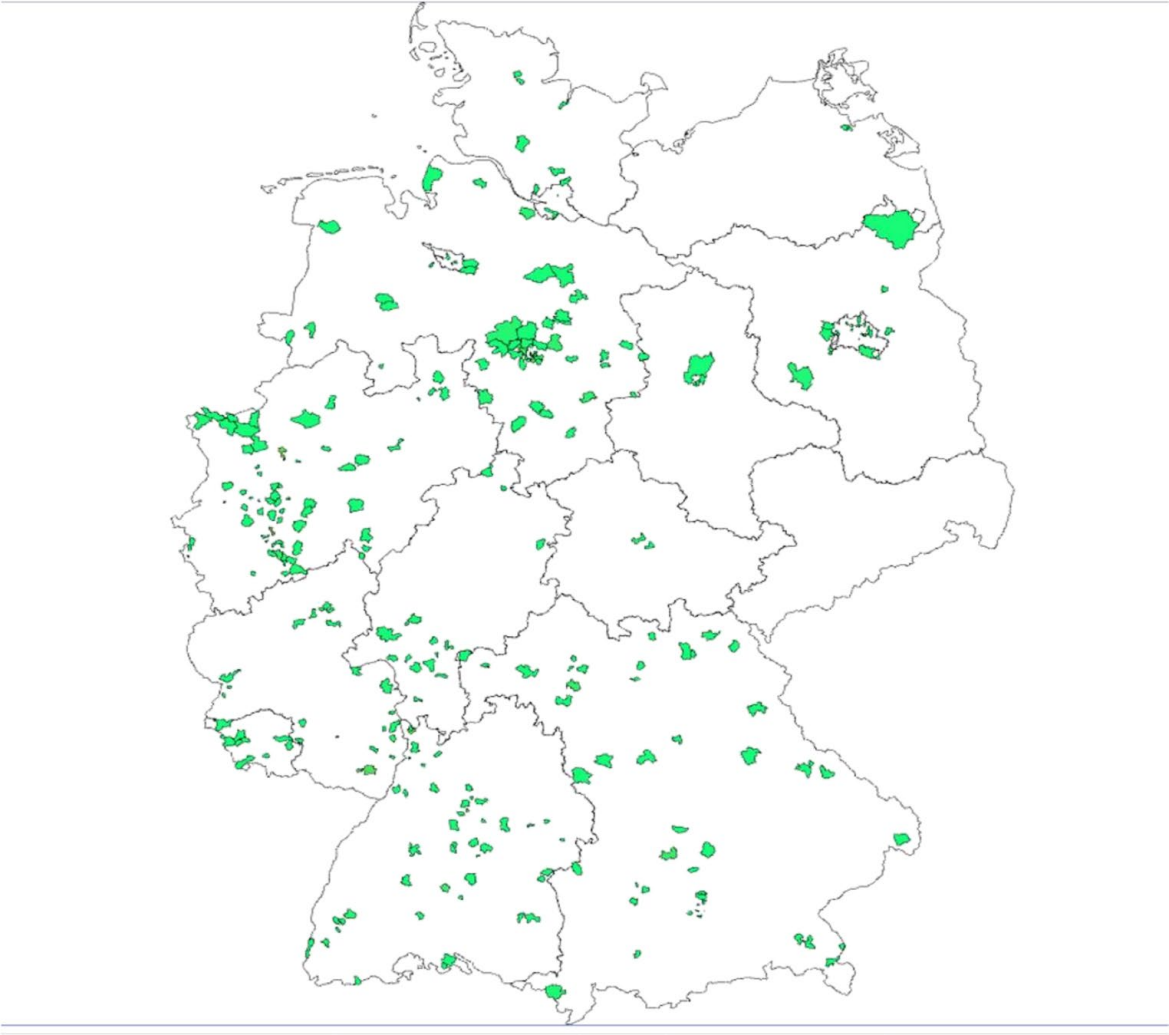
Geographical origin of captive chameleon faecal samples (*n* = 670) according to postcode districts in Germany

In total, 670 faecal samples of captive chameleons representing 25 species of different genera (e.g. *Chameleo*, *Furcifer*, *Triocerus*, *Calumma*, *Bradypodion*, *Rhampholeon*, *Kinyongia*, see Fig. 1) were sent to exomed GmbH laboratory, Marburg, Germany (Table 1) between 2012 and 2019 and examined coprologically. Of these, 573 (85.8%) came from private owners, 67 (10%) from veterinary surgeons, and 30 (4.5%) from zoological gardens across Germany (Fig. 2). Each faecal sample was accompanied by a submission form stating the signalment of the animal (i.e. species, sex, weight, and age) and a short anamnesis (husbandry type, previous parasitological diagnoses, and antiparasitic treatments, see Online Resource 1). However, some information was incomplete due to missing details from the owners. In cases where the chameleon species was not specified, samples were recorded as “unknown species”. Similarly, sex and age were noted as unknown when not provided. The freshness of the samples could not be determined, as the time between sample collection and arrival at the laboratory was not documented. Due to the unknown freshness of the samples, some parasite stages may have continued to develop—or not—before processing, which can complicate morphological identification. In cases where species-level identification was uncertain, eggs or larvae were grouped into broader taxonomic categories.

**Table 1:** Number and origin of chameleon fecal samples (total *n* = 670)

Chameleon species		Common name	No. examined			Origin (private/ vet / zoo)
*Furcifer pardialis*		Panther chameleon	250			223/16/11
*Chamaeleo calyptratus*		Veiled chameleon	232			200/30/2
unknown species			103			84/14/5
*Calumma parsonii*		Parson’s chameleon	23			12/0/11
*Trioceros melleri*		Meller’s chameleon	12			11/0/1
*Chamaeleo dilepis*		Flap-necked chameleon	10			9/1/0
*Furcifer lateralis*		Carpet chameleon	6			6/0/0
*Trioceros jacksonii*		Jackson’s chameleon	6			6/0/0
*Furcifer oustaleti*		Malagasy giant chamaleon	5			5/0/0
*Trioceros hoehnelii*		Höhnels chameleon	5			4/1/0
*Triceros rudis*		Coarse chameleon	3			3/0/0
*Bradypodion thamnobates*		Natal Midlands dwarf chameleon	2			2/0/0
*Bradypodion fischeri*		Fischer’s chameleon	2			0/2/0
*Trioceros Bitaeniatus*		Side-striped chameleon	2			2/0/0
*Trioceros j. xantholophus*		Yellow-crested Jackson’s chameleon	2			2/0/0
*Furcifer verrucosus*		Warty chameleon	1			0/1/0
*Kinyongia boehmei*		Böhme’s two-horned chamaleon	1			1/0/0
*Rampholeon acuminatus*		Nguru pygmy chameleon	1			0/1/0
*Bradypodion setaroi*		Setaro’s dwarf chameleon	1			1/0/0
*Trioceros johnstoni*		Johnston’s chameleon	1			0/1/0
*Trioceros sternfeldi*		Crater Highlands side-striped chameleon	1			1/0/0
*Trioceros ellioti*		Elliot’s chameleon	1			1/0/0

To identify different parasites and their stages, direct saline fecal smears (DSFS) were performed according to Cooper (2009). For DSFS preparation, faeces (10 g) were mixed at a ratio of 1:1 with 0.9% NaCl solution to a uniform suspension of the sample, placed on a microscope slide, covered with a coverslip (22 × 22 mm; Nunc) and examined by a light microscope (100x or 400x magnification, Axio Imager M1^®^, Zeiss, Jena). Oxyurid, heterakid, ascarid, trematode and pentastomid eggs as well as protozoan oocysts, cysts and trophozoites were identified based on previous description guidelines (Barnard and Upton 1994; Wilson and Carpenter 1996; Schneller and Pantchev 2008; Pantchev 2011; Hallinger et al. 2019). *Strongyloides* spp., *Rhabdias* spp., and other thin-shelled nematode eggs could not be reliably identified based on morphology alone, so they were grouped together under the category thin-shelled nematode eggs, representing a limitation due to the absence of molecular confirmation. Sporulated oocysts were differentiated morphologically based on previous descriptions of *Isospora* spp., *Isospora jaracimrmani*, and *Choleoeimeria* spp. (Modrý and Koudela 1995; Modrý et al. 2000, 2001; Sloboda and Modrý 2006; Koudela and Modrý 2013). Faecal samples were listed as positive (Table 2), if at least one stage of the above-described endoparasites posing a health-compromising risk for chameleons or other lizards was diagnosed. Samples lacking any pathogenic parasitic stages or showing non-pathogenic genera (e.g. *Nyctotherus*) as well as pseudoparasites, were here classified as negative.

**Table 2:** Parasitological findings in chameleon faecal samples [(total: *n* = 670; positive for parasite stages: 223/670 (33.3%); negative: 447/670 (66.7%)]

Parasite species	No. positive samples	Host species
Oxyurid nematodes (Pharyngodonidae*)*	95/670 (14.1%)	*C. calyptratus* (61) *F. pardalis* (16) Unknown species (15) *C. parsonii* (2) *R. acuminatus* (1)
Coccidia (e.g. *Isospora jaracimrmani*, *Isospora* spp., *Choloerimeria* spp.)	53/670 (7.8%)	*F. pardalis* (25) *C. calyptratus* (19) Unknown species (5) *C. dilepsis* (2) *F. lateralis (1)* *T. jacksonii* (1)
Flagellated protozoa (e.g. *Leptomonas* spp., *Tritrichomonas* spp., *Proteromonas* spp.)	42/670 (6.3%)	*F. pardalis* (13) *C. calyptratus* (11) Unknown species (9) *C. dilepis* (3) *T. melleri* (3) *C. parsonii* (1) *F. oustaleti* (1) *T. jacksonii* (1)
Heterakids	24/670 (3.6%)	*F. pardalis* (7) *C. parsonii* (7) Unknown species (4) *F. oustaleti* (3) *C. calyptratus* (2) *B. thamnobates* (1)
Thin-shelled nematode eggs (e.g. *Strongyloides* spp., *Rhabdias* spp.)	24/670 (3,6%)	Unknown species (7) *C. calyptratus* (5) *F. pardalis* (5) *T. hoehnelii* (3) *T. j. xantholophus* (2) *T. melleri* (1) *T. rudis* (1)
Trematode eggs (Diginea)	4/670 (0.6%)	*F. pardalis*: (3) *C. parsonii* (1)
Pentastomids	2/670 (0.30%)	*F. pardalis*: (1) *F. oustaleti* (1)
Ascarids	1/670 (0.15%)	*F. pardalis*: (1)
Amoeba	1/670 (0.15%)	*F. pardalis*: (1)
Physalopteroidea	1/670 (0.15%)	*F. oustaleti*: (1)

### Chameleon necropsies

A total of 31 chameleon individuals (*n* = 31) were necropsied at exomed GmbH laboratory (Table 4). In these cases, the signalement (i.e. species, age, sex) and basic medical history, [e. g. clinical signs, husbandry, food, cause of death (natural death/euthanization by veterinary surgeons)] was reported (Online Resource 2). Additionally, tissue samples were collected and fixed in formalin for pathohistological examinations as previously described (Stacy 2021). In detail, the chameleon necropsy was performed on its body side by an incision along the ventral midline from the cloaca to the forelimb and shoulder blade and up to the dorsal side. By this incision procedure, the coelomic cavity was exposed without obstructions (Farris et al. 2015). Primarily, the gastrointestinal tract was visually inspected for the presence of metazoan endoparasites. Additionally, intestinal content was collected for DSFS. For metazoan endoparasite diagnosis, morphological identification was performed in accordance to Pantchev (2011) and Hallinger et al. (2019) using a light microscope supplied with a digital camera (Axio Imager M1^®^, Zeiss, Jena).

### Microbiological examination of necropsies

For further microbial identification, faeces and/or coelom swabs were taken from necropsied animals. For bacterial and fungal cultivation in vitro, both the Columbia/MacConkey sheep blood agar (5%) as well as the Sabouraud dextrose agar (SDA) were used (BioMerieux, Charbonnier les Bains, France). In addition, bacterial isolates were identified by gram staining, oxidase, and catalase tests (BioMerieux, Charbonnier les Bains, France), together with a commercially available API 20 E/NE kit (BioMerieux, Charbonnier les Bains, France), as formerly described in reptiles (Marenzoni et al. 2015; Hallinger et al. 2019).

### Statistical analysis (general χ² tests)

Associations between parasite prevalence and host factors (species, age class or sex) were assessed using χ² tests of independence. For each analysis, expected counts for each category were calculated as (row total × column total) ÷ grand total. Only groups with sufficient sample sizes to meet expected frequency assumptions were included in statistical tests; rare species or categories with very low counts were excluded but are reported in descriptive tables for transparency. Statistical significance was determined at α = 0.05. All analyses were performed using GraphPad Prism (version 10, GraphPad Software, San Diego, CA, USA).

## Results

### Faecal samples

**Fig. 3:**
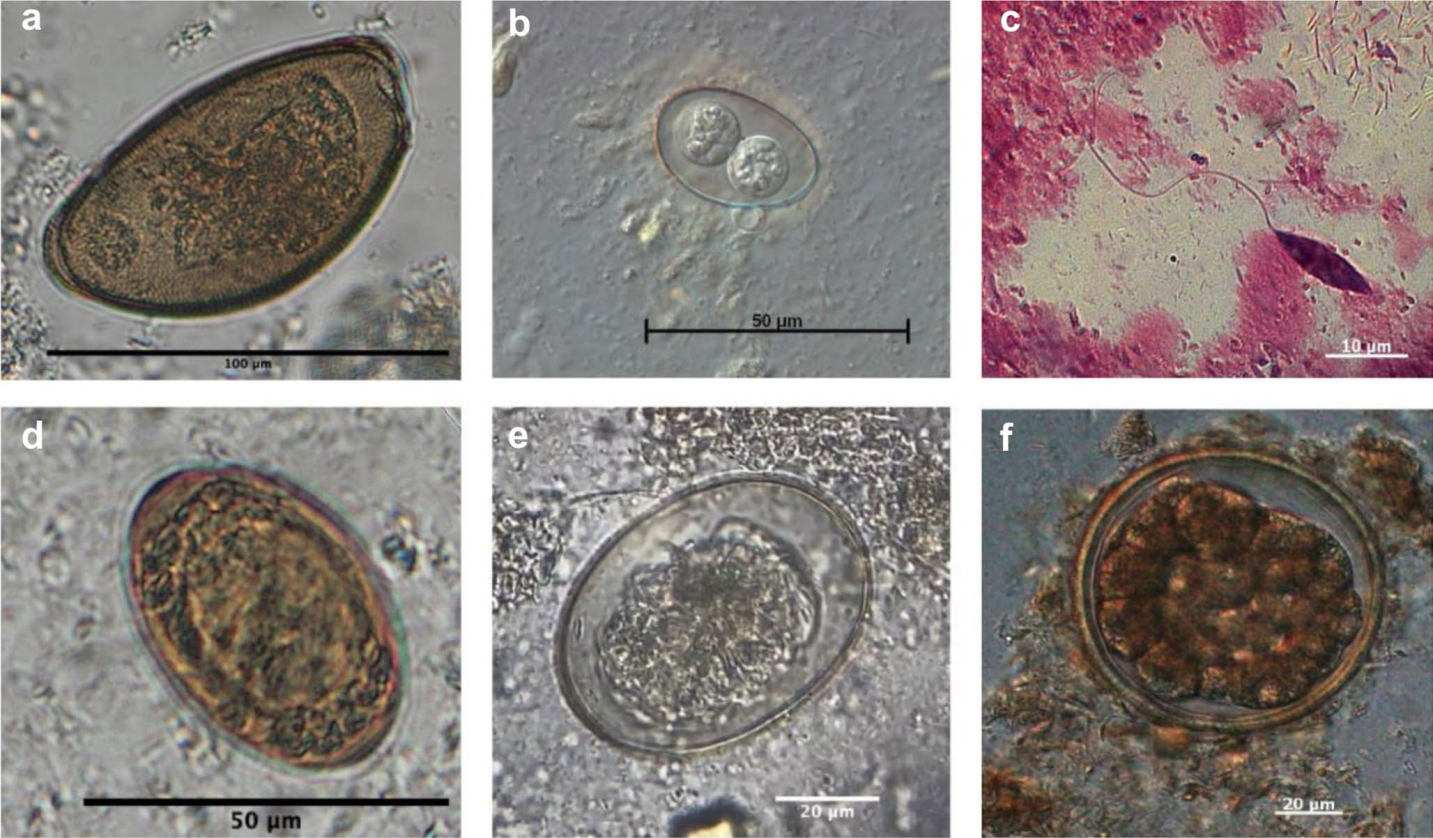
Exemplary photomicrographs of different parasitic stages found in chameleon faecal samples. (**a**) operculated oxyurid egg (Veiled chameleon), (**b**) sporulated *Isospora* spp. oocyst containing two sporocysts with four sporozoites each (Veiled chameleon), (**c**) Trichrome stained flagellated *Leptomonas* spp. trophozoites (Meller’s chameleon), (**d**) trematode egg with miracidium (Jackson’s chameleon), (**e**) embryonated pentastomid egg with larva (Malagasy giant chameleon), (**f**) *Hexametra angusticaecoides* egg (Panther chameleon)

**Fig. 4:**
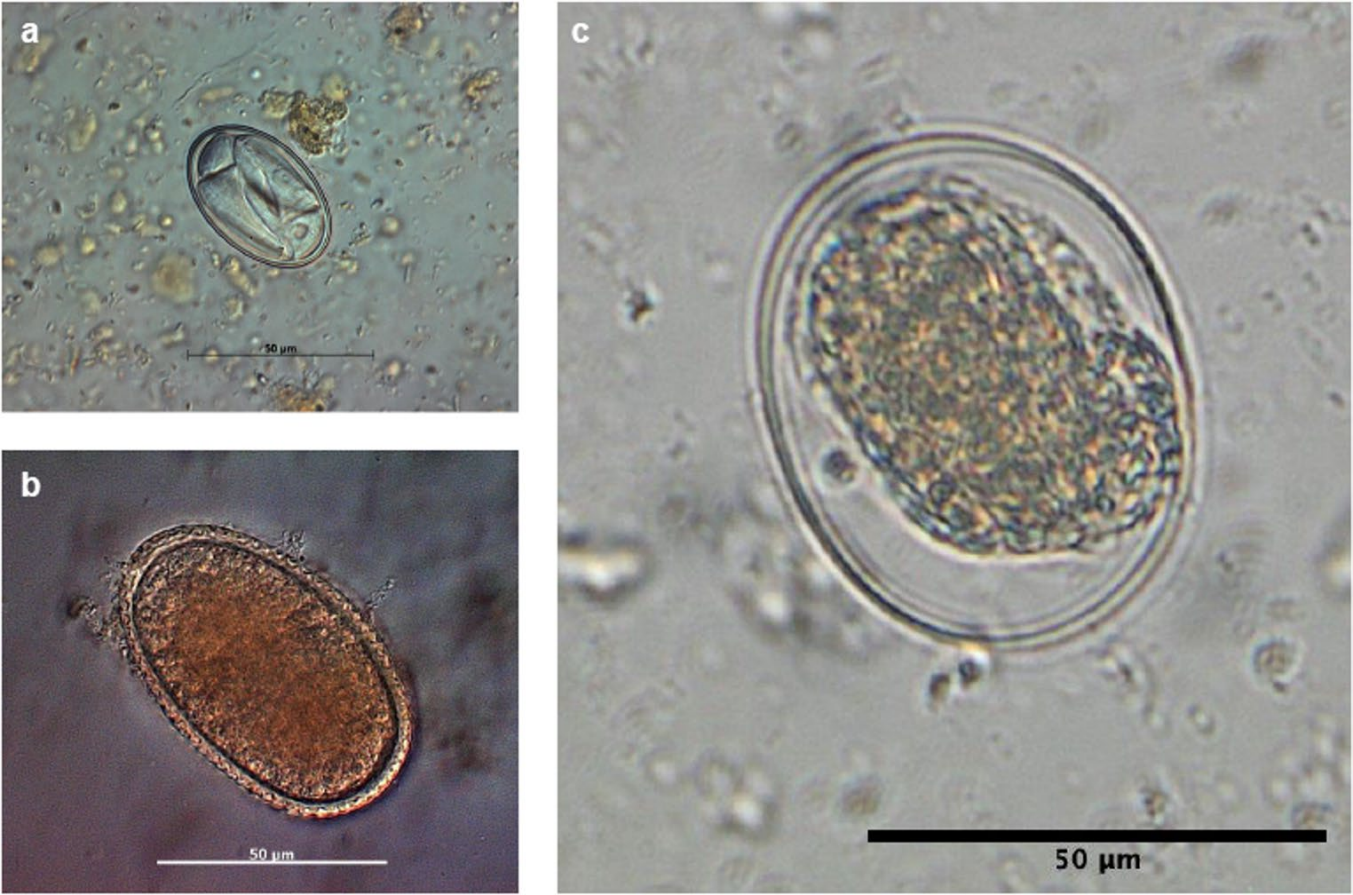
Selected photomicrographs of nematode eggs found in chameleon faecal samples. (**a**) spirurid egg containing a larva (Malagasy giant chameleon), (**b**) unembryonated heterakid egg (Veiled chameleon), (**c**) thin-shelled nematode egg with a morula stage (Panther chameleon)

DSFS-based coproscopical analyses of pet chameleon faecal samples from Germany revealed a wide spectrum of gastrointestinal protozoan and metazoan parasites. In total, 33.2% (223/670) faecal samples proved positive for parasite infections. Among the positive samples from DSFS testing the following parasitic stages were diagnosed: oxyurid eggs (14.1%), coccidian oocysts (7.8%), flagellated protozoan trophozoites (6.3%), heterakid eggs (3,6%), thin-shelled nematode eggs (3,6%), trematode eggs (0.6%), pentastomid eggs (0.3%), ascarid eggs (0.15%), *Entamoeba* cysts (0.15%) and *Physalopteroides* spp. eggs (0.15%) (please refer to Table 2). Representative photomicrographs are shown in Figs. 3 and 4. No cestode eggs were found in the current faecal samples.

**Fig. 5:**
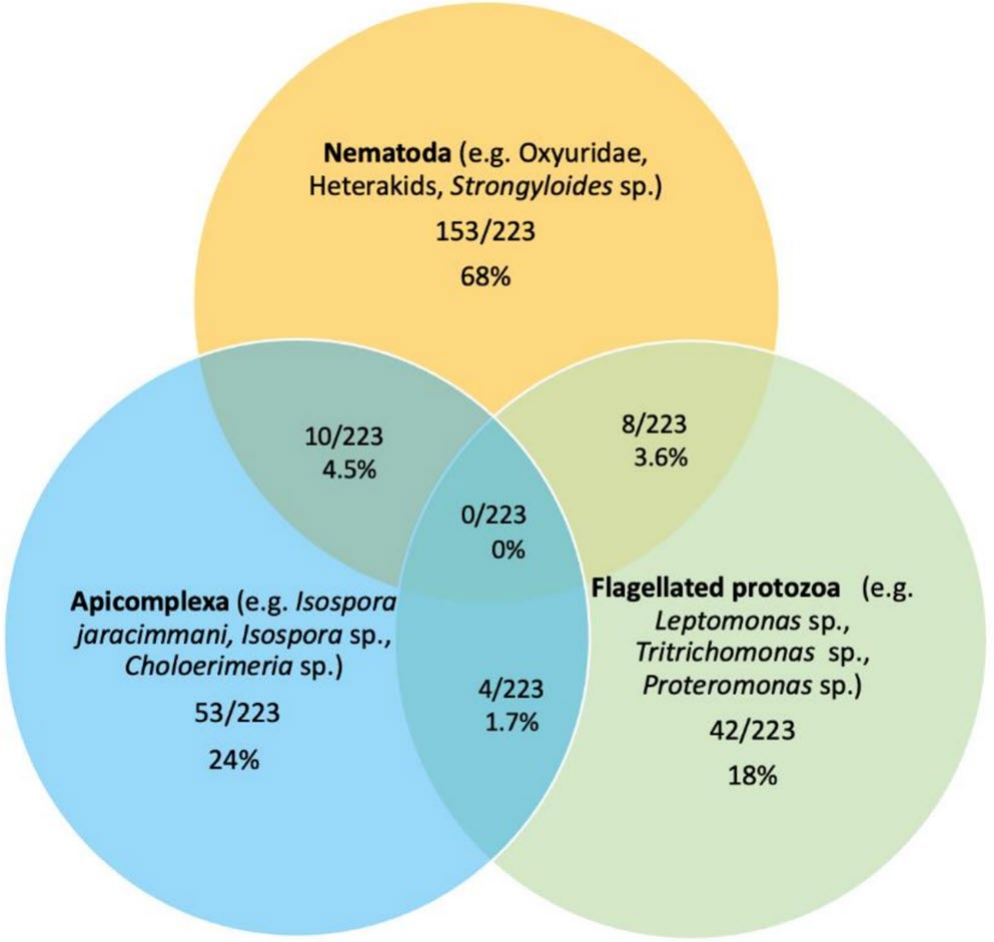
Endoparasite mono- and co-infections in faecal samples of chameleons (*n* = 670) kept in captivity in Germany

A total of 9.8% of the 223 parasite-positive chameleon samples showed co-infections (Fig. 5). Among these, 4.5% (10/223) of animals exhibited an ‘coccidia-nematode’ co-infection, while 3.6% (8/223) of chameleons had a ‘nematode-flagellated trophozoite’ co-infection. Additionally, 1.7% (4/223) of animals showed a ‘coccidia-flagellated trophozoite’ co-infection. Notably, in the faecal samples, no evidence of multiple infections of tri-, tetra- or higher-orders were detected.

Oxyurid nematodes were the most prevalent metazoan parasites with 14.1%. Interestingly, the oxyurid prevalence varied with the chameleon species, since it proved significantly higher in Veiled chameleons (*C. calyptratus*: 26.2%) when compared to Panther chameleons (*F. pardalis*) (6.4%) of this study (Chi-Square test: χ2 = 35.4, df = 1, *P* < 0.0001*)*. Overall, no significant correlation was found between parasite infection and either age (i.e. juvenile < 1 year of age versus adult > 1 year of age) or sex (Table 3).

**Table 3:** Prevalence of oxyurids in host species, with sex and age distribution (M=male/F=female/N.A.= not available/undetermined)

Species	*N*	Positive	Negative	Prevalence (%)	Sex (M/F/*N*.A.)	Age Class (Adult/Juvenile)
*C. calyptratus*	232	61	146	26.2	118/38/76	Adult/Juvenile
*F. pardarlis*	250	16	187	6.4	193/36/22	Adult/Juvenile
Unknown species	103	15	62	14.6	64/11/28	Adult/Juvenile
*C. parsonii*	14	2	12	14.2	7/2/5	Adult/Juvenile
*R. acuminatus*	1	1	0	100	0/0/1	N.A.

Other parasitic nematode stages like heterakid eggs (3.6%) and thin-shelled nematode eggs (e.g. *Strongyloides* spp., *Rhabdias* spp.) (3.6%) were found at a lower prevalence. Only one sample proved positive for ascarid eggs (0.1%) and another for *Physalopteroides* spp. eggs (0.1%). Intestinal trematode eggs were found in 4 samples (0.6%) whilst pentastomid eggs were present in two faecal samples (0.3%). When considering intestinal protozoan parasites, these revealed as the second most frequently diagnosed endoparasites. Among the protozoans, coccidia were the most prevalent in analyzed faecal samples. In this context, the genus *Isospora* showed the highest prevalence among the identified apicomplexans with 5.1% (34/670), whilst *Isospora jaracimrmani* (1.2%), *Choleoeimeria* spp. (0.3%) and un-sporulated apicomplexan oocysts (1.3%) were found at lower prevalences. *Isospora* spp. oocysts were ovoid, measuring 21–28 × 18–25 μm, with sporocysts measuring 12–13.2 × 9–12 μm. In contrast, *Isospora jaracimrmani* oocysts were larger and ellipsoidal to pyriform, measuring 36–41.6 × 24–28.7 μm, with sporocysts measuring 10.5–16.2 μm. Besides coccidia, *Entamoeba* cysts (0.15%) and cysts of intestinal flagellates (0.9%) were also diagnosed. Among the flagellated protozoa, *Leptomonas* spp. reached the highest prevalence (2.2%) followed by *Trichomonas* spp. (1.5%) whereas other flagellates like *Tritrichomonas* spp. (1.1%), *Proteromonas* spp. (0.3%) and *Monocercomonas* spp. (0.1%) ranked lower. We observed a statistically significant difference in the prevalence of coccidia between adult and juvenile animals since juveniles were more frequently affected (*p* < 0.0009).

### Chameleon necropsies

**Fig. 6:**
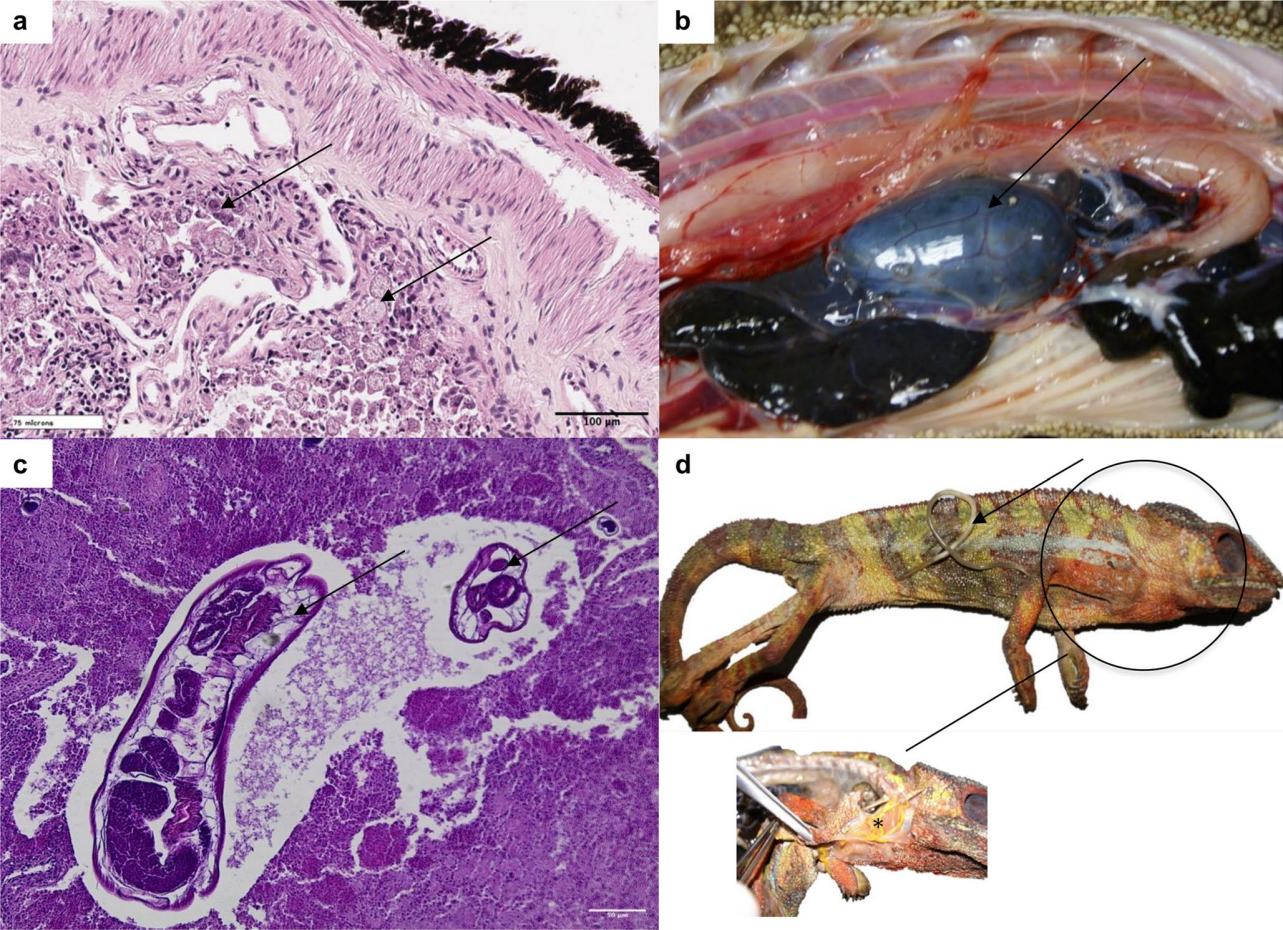
Exemplary images from chameleon necropsies and related histological findings. (**a**) Coccidian meronts and gamonts (black arrows) in the intestinal epithelium of a Veiled chameleon (400x magnification), (**b**) close-up of a dilated gallbladder (black arrow) caused by bile duct obstruction associated with a severe *Choleoeimeria* sp. infection, (**c**) cross section of a *Spinicauda* sp. nematode (black arrows) in the small intestine mucosa of a Panther chameleon (200x magnification), (**d**) adult *Hexametra angusticaecoides* nematode (indicated by black arrow) removed from the subcutaneous tissue, co-infected with *Foleyella furcata* in the serosa, exposed after removal of the skin (asterisk) of a Panther chameleon

A total of 31 animals originating from 10 different chameleon species were subjected to necropsy. Here, most animals (13/31, 41%) belonged to the Panther chameleon (*F. pardalis*) and other species (details of origin Table 1). In necropsies, 19 chameleons (61%) tested positive for endoparasite infections (Table 4). Pathological examination revealed that severe parasitoses were identified as the cause of death in at least 13 animals (40.6%), including isosporosis (*n* = 3; 9.3%) (Fig. 6a), choleoeimeriosis (*n* = 3; 9.3%) (Fig. 6b), heterakiosis (*n* = 2; 6.2%) (Fig. 6c), oxyuridosis (*n* = 1;3.1%). An animal (3.1%) died due to a tetra-parasitic infection showing leptomonosis, trichomonosis, foleyellosis (*Foleyella furcata*) and hexametrosis (Fig. 6d). One Jackson’s chameleon (*T. jacksonii*) died due to severe protozoa-driven enteritis caused by *Leptomonas* spp. and *Trichomonas* spp. infections. Furthermore, there were five cases of co-infections (*n* = 5, 15.6%).

**Table 4:** Parasitological findings in chameleon necropsies [(total: *n* = 31; positive for parasite stages: 19/31 (61.2%)]

Parasite Species	No. of positive (%)	Host species *n*.
Flagellated protozoa (e.g. *Leptomonas* spp., *Tritrichomonas *spp., *Proteromonas* spp.)	Total: 6 (19.3)	*F. pardalis* (3) *C. calyptratus* (1) *T. melleri* (1) *T. jacksonii xantholopus *(1)
*Isospora* spp.	Total: 5 (16.1)	*C. calyptratus* (1) *F. pardalis* (3) *T. melleri* (1)
Oxyurid nematodes (Pharyngodonidae)	Total: 4 (12.9)	*C. calyptratus* (4)
*Choleoeimeria *spp.	Total: 3 (9.6)	*F. pardalis* (1) *T. melleri* (2)
Heterakids (e.g. *Spinicauda* spp.)	Total: 2 (6.4)	*F. pardalis *(1) *B. thamnobates *(1)
*Hexametra angusticaecoides*	Total: 1 (3.2)	*F. pardalis* (1)
*Foleyella furcata*	Total: 1 (3.2)	*F. pardalis* (1)
Thin shelled nematode eggs	Total: 1(3.2)	*Calumma parsonii* (1)

Other etiological causes identified for chameleon death were gout (15.6%), mycosis (9.37%), salmonellosis (9.37%), other bacterial infections (9.37%), intestinal obstipation (6.25%), mechanical trauma (6.25%), adenocarcinoma (6.25%), malnutrition (3.1%) and hypervitaminosis D3 (3.1%). In two chameleons, the cause of death remained unknown even though showing enteritis and hepatitis (see Online Resource 2).

## Discussion

The present large-scale epidemiological study on gastrointestinal parasites of chameleons kept in captivity in Germany reveals a high diversity of protozoan and metazoan endoparasite species, thereby confirming findings from previous prevalence studies (Pantchev 2011; Biallas 2013).

Referring to chameleon oxyurid species, *Parapharyngodon kenyaensis* (Goldberg and Bursey 2008), *Pharyngodon dimorpha* and *Thelandros meridionalis* (Brygoo 1963) have been so far described in wild- and captivity-kept chameleons (Okulewicz et al. 2015; Eckhardt et al. 2019; Stets 2019). In the current study, oxyuridosis represented the most frequently found parasitosis (14.1%). However, earlier publications reported on a considerably higher prevalence (27.3% in Pantchev 2011 and 25.5% in Biallas 2013). This might reflect an increasing awareness among owners leading to a more frequent testing for gastrointestinal parasite infections and subsequent treatment in the last years. Among the various chameleon species assessed for oxyuridosis, we observed a notable difference in prevalence. Hence, *C. calyptratus* showed a significantly higher oxyurid prevalence than *F. pardalis.* This finding may be explained by its overrepresentation in the sample pool due to its broad availability and low purchase costs, unfortunately often resulting in suboptimal husbandry conditions caused by inexperienced owners. In contrast, other, more expensive species like the Panther chameleon are typically kept by experienced owners, which may also be more aware on regular monitoring measures for detection of parasitic infections.

In contrast to captivity, the chances of reinfections are lower in the wild, and a delicate equilibrium between reptile hosts and their oxyurid species has been described. Thus, certain oxyurid nematodes efficiently support the intestinal microbiome to keep the animal in balance (Schneller and Pantchev 2008; Hallinger et al. 2018; Wellehan and Walden 2019; Eckhardt et al. 2019). In captivity, however, chameleons are confined to limited space and often suffer from suboptimal husbandry conditions, disrupting this equilibrium and leading to massive infections with these monoxenous tenacious parasites, finally resulting in (sub)clinical disease (Schneller and Pantchev 2008; Rinaldi et al. 2012; Pantchev 2013; Hallinger et al. 2019). Oxyuridosis in chameleons is usually of low pathogenicity, though severe infections can cause malabsorption, weight loss and nonspecific signs, especially in young, gravid or immunocompromised animals (Pantchev 2011, 2013; Biallas 2013; Hallinger et al. 2018; Kubiak 2020). In the current study, oxyurid mono-infection was restricted to solely one necropsied Veiled chameleon, however this parasitosis was accompanied by bacterial co-infections with *Salmonella enterica* and *Stenotrophomonas maltophila*. Consequently, it is challenging to attribute the cause of death solely to oxyuridosis. This assumption aligns well with former findings on low intrinsic pathogenicity of oxyurids, reporting that mono-infections did not reveal as single cause of death (Biallas 2013).

Because most thin-shelled nematode eggs in assessed chameleons could not be identified to the species level, this group likely includes both *Strongyloides* spp. and *Rhabdias* spp. nematode species which are widespread parasites affecting captive and wild animals, including chameleons (Hallinger et al. 2020; Biallas 2013). *Strongyloides* spp. infections may cause both respiratory and gastrointestinal signs due to larval migration and can be fatal (Schneller and Pantchev 2008; Biallas 2013; Heng et al. 2023). *Rhabdias* spp. are lung-dwelling nematodes infecting a range of reptiles and amphibians (Frank 1985). Several species have been described from wild chameleons and differentiated to species level (Lhermitte-Vallarino et al. 2008, 2009, 2011), whereas in captive chameleons, infections have only sporadically been reported and typically identified as *Rhabdias* spp. (Biallas 2013). Pulmonary infections with *Rhabdias* spp. may lead to respiratory symptoms, but this depends on the infection grade (Frank 1985).

The prevalence of heterakids and ascarids in the current study was relatively low, when compared with previously recorded prevalences of 6–7% in chameleons (Pantchev 2009; Biallas 2013). *Spinicauda sonsinoi* (Brygoo 1963) and *Spinicauda inglisi* (Chabaud and Brygoo 1962) are the most common heterakid species in Malagasi chameleons. These parasites show a rather low pathogenicity if the parasitic burden is low (Schneller and Pantchev 2008). However, ascarid species like *Hexametra angusticaecoides* have high pathogenic potential and severe hexametriosis often leads to animal death (Schneller and Pantchev 2008; Pantchev 2011; Barton et al. 2020; Reitl et al. 2021). Of note, *H. anguisticaecoides* has a dual transmission mode in chameleons, an indirect infection route via consumption of *H. angusticaecoides*-infected arthropod intermediate hosts and a direct transmission route by the ingestion of embryonated eggs from the environment (Chabaud et al. 1962; Reitl et al. 2021). Remarkably, this ascarid nematode has been reported to infect at least two different chameleon species (i.e. *F. pardalis* and *C. calyptratus*) without involvement of intermediate hosts (Coke 1997). Referring to clinical signs, larval migration-driven subcutaneous swellings are commonly reported in chameleons (Pantchev 2013; Reitl et al. 2021).

The low prevalence of other helminths such as filarial nematodes (Spirurida), might be explained by the indirect developmental cycle of filarial parasites which involves hematophagous arthropod vectors and has only been observed in wild-caught individuals (Pantchev 2011; Biallas 2013 Pellett et al. 2014). *Foleyella furcata* is the species most frequently described in clinical cases. (Szell et al. 2001; Pellet et al. 2014; Maia et al. 2014). Additionally, spirurid nematodes, specifically from the genus *Physaloptera*, are infrequently seen in captive chameleons (Pantchev 2011). In literature, no clinical cases of physalopterosis in chameleons have been described so far.

Trematode eggs were only detected in four samples, indicating an exceptionally low occurrence. To date, two species of digenean trematodes have been described in chameleons, i.e. *Postorchigenes* spp. and *Malagashitrema* spp. (Morsy et al. 2012).

Concerning coccidian parasites in chameleons, the genus *Isospora* is represented by ten species, which have been identified in both wild- and captivity-kept animals (Brygoo 1963; Modrý et al. 1997, 2000, 2001; Abdel-Wasae 2004; McAllister 2012; Ekawastasi et al. 2021). Moreover, nine tetra-sporocystic coccidian species (i.e. *Eimeria* spp. and *Choleoeimeria* spp.) have been characterized within the Chamaleonidae family (Sergent 1902; Modrý et al. 2001; b; Sloboda and Modrý 2006). In the current study, the coccidia prevalence (7.8%) was lower compared to oxyurids (14.1%). However, current necropsies revealed a higher level of mortality driven by coccidiosis (due to *Isospora* spp. or *Choleoeimeria* spp. infections) when compared to oxyuridosis indicating a higher pathogenicity of these protozoa. Hence, six animals exhibited severe intestinal mucosal damage caused by massive coccidian intracellular replication leading to a massive presence of meront- and oocyst stages in the small intestine lumina. Here, coccidia prevalence reached 7.8%, a level similar to findings reported previously by Pantchev (2011) with 10.6%. In contrast, Biallas (2013) recorded a much higher prevalence with 21.7% in a smaller sample size. Eventually, this discrepancy may be explained by a higher proportion of juveniles in former study, the lack of prior antiparasitic treatment or other, unassessed factors like housing and hygiene conditions. As expected, juvenile chameleons (3.7%) showed a higher rate of coccidian infections than adults (2.5%) in the current study. This age-related susceptibility for intestinal coccidia infections is in line with previous reports from Biallas (2013) and Pantchev (2011) and is also commonly recorded for mammalian hosts (Foreyt 1990; Bangoura and Bardsley 2020).

Interestingly, coccidiosis frequently occurred in co-infections. Hence, co-infections of *Isospora* spp. and oxyurids were the most common in the current study, thereby agreeing with findings of Biallas (2013) and re-confirming that these parasitic infections represent the two most prevalent ones in captive reptile husbandry, as both parasites have direct life cycles and short prepatent periods (Walden and Mitchell 2021; Pike et al. 2023). Of note, captive-bred chameleons like *C. calyptratus* and *F. pardalis* revealed a twice higher coccidia prevalence compared to their wild-caught counterparts; a phenomenon attributed to the aforementioned factors.

Reptilian coccidiosis, like in mammals and birds, is a self-limiting disease (Long 1982; Quiroz-Castañeda and Dantán-González 2015; Hallinger et al. 2019). While typically being asymptomatic and self-limited in the wild, captivity settings allow for reinfections with varying degrees of severity, depending on various extrinsic and intrinsic factors (Mutschmann 2006; Bangoura and Bardsley 2020). Furthermore, infections with coccidian parasites have a higher likelihood of causing mortality and morbidity in captive lizards (Modrý and Sloboda 2004). During *Isospora* infections of the intestinal mucosa, a wide spectrum of clinical signs may be observed, ranging from watery to hemorrhagic diarrhea, to general symptoms like anorexia, lethargy, weight loss, cachexia, and, in some cases, even gut invagination and obstipation (Mutschmann 2006; Schneller and Pantchev 2008; Biallas 2013). In contrast, *Choleoeimeria* species exclusively parasitize the epithelial mucosa of the gallbladder, eventually causing cholecystitis with epithelial cell destruction and gall bladder obstruction with similar symptoms as mentioned before (Modrý and Sloboda 2004).

Most enteric flagellated protozoa bear low pathogenicity and are often referred to as opportunistic parasites since the development of related clinical symptoms is multifactorial and based on several parameters like stress, overcrowding, nutritional deficiencies, and host specificity (Zwart et al. 1984; Mutschmann 2006). Nonetheless, in the case of *Leptomonas* spp., bloody colitis was consistently reported in parasitized chameleons (Schneller and Pantchev 2008; Pantchev 2011). Overall, a potential role of feeder insects as paratenic hosts for *Leptomonas* spp. infections in chameleons has been proposed (Bayon 1915; Wenyon 1920) but remains unconfirmed. In the current study, most animals with leptomonosis suffered from enteritis but additionally showed co-infections with other parasites. Moreover, the presence of flagellated *Leptomonas* trophozoites in the gut lumen was mostly linked to intestinal inflammation in vivo.

In the current study, *Entamoeba* stages were diagnosed in only one sample, thereby emphasizing the rareness of this infection in captive chameleons. Nevertheless, *Entamoeba invadens* is widely recognized for its high pathogenicity in snakes and lizards, but only a single case of amoebiasis has been reported in a chameleon co-infected with *Mycobacterium simiae* complex (Barnard and Upton 1994; Schneller and Pantchev 2008; Wellehan and Walden 2019; Fuentes et al. 2023). Thus, the real impact of dysenteric amoebiasis in chameleons remains uncertain, rendering prevention essential for health sustainment (Barnard and Upton 1994; Hatt 2010).

Pentastomids have been recorded in a wide range of reptile species, including snakes, geckos, agamas, skinks, varanids and crocodiles (Deakins 1973; Paré 2008; Pantchev and Tappe 2011; Rataj et al. 2011). Importantly, certain pentastomid genera like *Armillifer* and *Porocephalus* own zoonotic potential, especially those parasitizing snakes (Pantchev and Tappe 2011; Wellehan and Walden 2019; Hallinger et al. 2020). Lizards are the most common final hosts for the genus *Raillietiella* (Paré 2008; Barton 2009; Mendoza-Roldan et al. 2022), which can also infect chameleons (Sapion-Miranda et al. 2025; Hellebuyck et al. 2025). The zoonotic potential of *R. orientalis* has not been confirmed yet, however, one case report diagnosed *Raillietiella* sp. larvae in a human (Tappe et al. 2016). Even though reflecting a rare event, this case calls for further research on the zoonotic potential of pentastomids. These arthropod endoparasites own a highly complex, malunderstood lifecycle that may involve or not intermediate hosts (Bosch 1986). Cockroaches serve as intermediate hosts for *Raillietiella* spp., raising severe concerns on parasite transmission in regions where these synanthropic insects commonly occur in household or zoos (Lavoipierre and Lavoipierre 1965; Lavoipierre and Rajamanickam 1973; Ali et al. 1985; Bosch 1986; Palmisano et al. 2022).

Notably, neither intestinal cestode adults, proglottids nor cestode eggs were found in this parasitological study.

## Conclusion

In recent years, keeping chameleons as exotic pets has become more popular in Germany and other countries. Their fascinating phenotype and the chance of housing in small-spaced terraria renders chameleons as attractive pets for apartment dwellers. However, responsible breeding practices and appropriate husbandry conditions are essential to maintain animal health and to control parasite infections. This includes a routine maintenance of clean enclosures, monitoring for parasite infections by regular coproscopy and the implementation of effective parasite control measures, whenever necessary. Regular medical examinations are essential to maintain the overall health and resilience of captive chameleon populations. Moreover, it seems essential to monitor and control wild-caught reptile imports and to assess their infection status, especially with respect to zoonotic parasites (e.g. pentastomids). In addition, the potential role of arthropods as mechanical vectors or intermediate hosts needs to be further investigated to better understand their role in reptile parasite epidemiology. Overall, this study offers a base line dataset that will provide not only veterinarians but also herpetologists with valuable insights into the neglected field of reptile medicine.

## Supplementary information

Below is the link to the electronic supplementary material.

Supplementary File 1 (XLSX 163 KB)

Supplementary File 2 (XLSX 30.6 KB)

## Data Availability

The datasets generated and analysed during the current study are available as supplementary material accompanying this article.

## References

[CR1] Abdel-Wasae BM (2004) *Isospora taizii* (Apicomplexa: Eimeriidae), a new coccidian parasite from the Yemen chameleon (*Chamaeleo calyptratus*) (Sauria: Chamaeleonidae) in Taiz City, Yemen Republic. Assiut Univ Bull Environ Res 7(2):29–34

[CR2] Ali JH, Riley J, Self JT (1985) A review of the taxonomy and systematics of the pentastomid genus *Raillietiella* Sambon, 1910 with a description of a new species. Syst Parasitol 7(2):111–123. 10.1007/BF00009814

[CR3] Bangoura B, Bardsley KD (2020) Ruminant coccidiosis. Vet Clin North Am Food Anim Pract 36(1):187–203. 10.1016/j.cvfa.2019.12.006 32029184 10.1016/j.cvfa.2019.12.006

[CR4] Barnard SM, Upton SJ (1994) A veterinary guide to the parasites of reptiles: Protozoa. Krieger Publishing Company, Malabar (FL)

[CR5] Barton D (2009) Pentastomid parasites of the introduced Asian house gecko, *Hemidactylus frenatus* (Gekkonidae), in Australia. Comp Parasitol 74:254–259. 10.1654/4209.1

[CR6] Barton DP, Martelli P, Luk W, Zhu X, Shamsi S (2020) Infection of *Hexametra angusticaecoides* Chabaud & Brygoo, 1960 (Nematoda: Ascarididae) in a population of captive crested geckoes, *Correlophus ciliatus* Guichenot (Reptilia: Diplodactylidae). Parasitology 147(6):673–680. 10.1017/S0031182020000219 32046802 10.1017/S0031182020000219 PMC10317624

[CR7] Bayon H (1915) Herpetomonidae found in *Scatophaga hottentota* and *Chamaeleon pumilus*. Trans R Soc S Afr 61:1–3

[CR8] Biallas S (2013) Zur Bedeutung von Endoparasiten bei Chamäleons (Sauria: Chamaeleonidae) aus Wildfängen und Nachzuchten. Dissertation, Veterinärmedizinische Fakultät der Universität Leipzig

[CR9] Bosch H (1986) Experimental life-cycle studies of *Raillietiella* Sambon, 1910 (Pentastomida: Cephalobaenida): the fourth-stage larva is infective for the definitive host. Z Parasitenkd 72(5):673–680. 10.1007/BF00925489

[CR10] Bower DS, Brannelly LA, McDonald CA, Webb RJ, Greenspan SE, Vickers M, Gardner MG, Greenlees MJ (2019) A review of the role of parasites in the ecology of reptiles and amphibians. Austral Ecol 44(3):433–448. 10.1111/aec.12695

[CR11] Brygoo ER (1963) Contribution to the knowledge of the parasitology of Madagascan chameleons. Ann Parasitol Hum Comp 38:149–336 14076079

[CR12] Carpenter A, Rowcliffe M, Watkinson A (2004) The dynamics of the global trade in chameleons. Biol Conserv 120:291–301. 10.1016/j.biocon.2004.03.002

[CR15] Cervone M, Fichi G, Lami A, Lanza A, Damiani GM, Perrucci S (2016) Internal and external parasitic infections of pet reptiles in Italy. J Herpetol Med Surg 26(3–4):122–130

[CR14] Chabaud AG, Brygoo ER (1962) Nématodes parasites de caméléons malgaches—Deuxième note. Ann Parasitol Hum Comp 37(4):Article 4. 10.1051/parasite/1962374569

[CR13] Chabaud AG, Brygoo ER, Petter AJ (1962) Parasitologie-demonstration experimentale de differents types de cycles evolutifs potentiels chez un ascaride de cameleon. C R Hebd Seances Acad Sci 255(18):2320 14019820

[CR16] Coke RL (1997) Hexametra transmission between wild caught panther chameleons (*Chamaeleo pardalis*) and captive born veiled chameleons (*Chamaeleo calyptratus*): A case report. Proc Assoc Rept Amphib Vet 4:25–27

[CR17] Collicutt NB, Stacy NI, Walden HS, Childress A, Dill J, Anderson M, Wellehan JFX (2017) Infection with a novel derogenid trematode in a flap-necked chameleon (*Chamaeleo dilepis*). Vet Clin Pathol 46(4):629–634. 10.1111/vcp.12537 28817168 10.1111/vcp.12537

[CR18] Cooper JE (2009) In-practice and field techniques for the investigation of parasitic infections. J Exot Pet Med 18(4):289–298. 10.1053/j.jepm.2009.09.007

[CR19] Damiani G, Buggiani C, Perrucci S (2006) Coccidiosis and cryptosporidiosis in the veiled chameleon (*Chamaeleo calyptratus*) kept in captivity. Vet (Cremona) 20(2):63–66

[CR20] Deakins D (1973) Pentastome in captive reptiles. Dissertation. ProQuest. https://www.proquest.com/openview/dd78eaeb6fb93e44e3bfb695dc729e5d/1?pq-origsite=gscholar&cbl=18750&diss=y

[CR21] Diaz R, Anderson C, Baumann D et al (2015) Captive care, raising, and breeding of the veiled chameleon (*Chamaeleo calyptratus*). Cold Spring Harb Protoc 2015:943–949. 10.1101/pdb.prot087718 26310902 10.1101/pdb.prot087718

[CR22] Dillehay DL, Boosinger TR, MacKenzie S (1986) Gastric cryptosporidiosis in a chameleon. J Am Vet Med Assoc 189(9):1139–1140 3505954

[CR23] Eckhardt F, Strube C, Mathes KA et al (2019) Parasite burden in a short-lived chameleon, *Furcifer labordi*. Int J Parasitol Parasites Wildl 10:231–240. 10.1016/j.ijppaw.2019.09.010 31667086 10.1016/j.ijppaw.2019.09.010 PMC6812308

[CR24] Ekawastasi I, Kitawka K, Domae H et al (2021) Phylogenetic characterization of *Isospora jaracimrmani* oocysts from a veiled chameleon (*Chamaeleo calyptratus*) reared at a zoo in Ishikawa, Japan. J Vet Med Sci 83(8):1240–1243. 10.1292/jvms.21-0152 34135210 10.1292/jvms.21-0152 PMC8437735

[CR25] Farris S, Squires M, Ridgley F et al (2015) Necropsies of reptiles: Recommendations and techniques for examining invasive species. Univ Fla Electron Data Inf Source. 10.32473/edis-uw382-2013

[CR26] Foreyt WJ (1990) Coccidiosis and cryptosporidiosis in sheep and goats. Vet Clin North Am Food Anim Pract, 6(3):655–670.10.1016/S0749-0720(15)30838-0 10.1016/s0749-0720(15)30838-0 2245367

[CR27] Frank W (1985) Infektion (Invasionen) mit Parasiten (Parasitosen). In: Isenbügel E, Frank W (eds) Heimtierkrankheiten. Ulmer. Stuttgart, Germany, pp. 226–320

[CR28] Fuentes EM, Rodríguez JP, Matute AR Chavarría LM (2023) Granulomatous pneumonia caused by *Mycobacterium simiae* complex and necroulcerative enteritis caused by *Entamoeba* spp in a panther chameleon (*Furcifer pardalis*). Conference abstract, Joint European Congress of Veterinary Pathology, Clinical Pathology and Comparative Pathology (ESVP–ECVP–ESVCP–ECVCP), Lisbon, Portugal

[CR29] Gałęcki R, Sokół R (2019) A parasitological evaluation of edible insects and their role in the transmission of parasitic diseases to humans and animals. PLoS ONE 14(7):e0219303. 10.1371/journal.pone.0219303 31283777 10.1371/journal.pone.0219303 PMC6613697

[CR31] Goldberg SR, Bursey CR (2008) Helminths from three species of African chameleons. Afr Zool 43(2):270–272. 10.1080/15627020.2008.11657243

[CR32] Hallinger MJ, Taubert A, Hermosilla C (2020) Occurrence of Kalicephalus, Strongyloides, and Rhabdias nematodes as most common gastrointestinal parasites in captive snakes of German households and zoological gardens. Parasitol Res 119(3):947–956. 10.1007/s00436-019-06526-0 31950252 10.1007/s00436-019-06526-0

[CR34] Hallinger MJ, Taubert A, Hermosilla C, Mutschmann F (2018) Occurrence of health-compromising protozoan and helminth infections in tortoises kept as pet animals in Germany. Parasites Vectors 11:352. 10.1186/s13071-018-2936-z 29914556 10.1186/s13071-018-2936-z PMC6006665

[CR33] Hallinger MJ, Taubert A, Hermosilla C, Mutschmann F (2019) Captive agamid lizards in Germany: Prevalence, pathogenicity and therapy of gastrointestinal protozoan and helminth infections. Comp Immunol Microbiol Infect Dis 63:74–80. 10.1016/j.cimid.2019.01.005 30961821 10.1016/j.cimid.2019.01.005

[CR35] Hatt JM (2010) Selected endoparasites in reptiles. Proc World Small Anim Vet Assoc (WSAVA) Congress, Geneva, Switzerland

[CR36] Heckmann RA (2014) Jackson’s chameleon (*Chamaeleo jacksonii*) infected with *Baylisascaris procyonis* (Nematoda). Proc Parasitol 57:73–85

[CR37] Hellebuyck T, Solanes-Vilanova F, Decorte B, Miljković J, Claerebout E (2025) Patent pulmonary infection with the invasive pentastomid *Raillietiella orientalis* in a panther chameleon (*Furcifer pardalis*) in Belgium. Animals 15(23):3433. 10.3390/ani15233433 41375490 10.3390/ani15233433 PMC12691265

[CR38] Heng Y, Sekar S, Panarese R, Hsu CD, Xie S (2023) Management of a fatal outbreak of strongyloidasis in a captive population of panther chameleons (*Furcifer pardalis*) with ivermectin. J Zoo Wildl Med 54(2):282–291. 10.1638/2022-0132 37428690 10.1638/2022-0132

[CR39] Koudela M, Modrý D (2013) Description of *Isospora jaracimrmani* sp. n. (Apicomplexa: Eimeriidae) from the Yemen chameleon *Chamaeleo calyptratus* (Sauria: Chamaeleonidae). Folia Parasitol 42(4):313–316 16898122

[CR40] Kubiak M (2020) Chameleons. In: Kubiak M (ed) Handbook of Exotic Pet Medicine, 1st edn. Wiley, pp 263–281. 10.1002/9781119389934.ch15

[CR41] Lavoipierre MMJ, Rajamanickam C (1973) Experimental studies on the life cycle of a lizard pentastomid. J Med Entomol 10(3):301–302. 10.1093/jmedent/10.3.301 4719296 10.1093/jmedent/10.3.301

[CR42] Lavoipierre MM, Lavoipierre M (1965) The American cockroach, *Periplaneta americana*, as an intermediate host of the pentastomid, *Raillietiella hemidactyli*. Med J Malaya 20(1):72–73 4221429

[CR45] Lhermitte-Vallarino N (2011) The lung nematode parasites of the genus Rhabdias (Rhabdiasidae): diversity and biology in the Chamaeleonidae (Squamata) and hypotheses on their evolution. Bull Soc Zool Fr 135(1-2):109–118

[CR43] Lhermitte-Vallarino N, Barbuto M, Ineich I et al (2008) First report of *Rhabdias* (Nematoda: Rhabdiasoidea) from lungs of montane chameleons in Cameroon: Description of two new species and notes on biology. Parasite 15(4):Article. 10.1051/parasite/2008154553 10.1051/parasite/2008154553 19202762

[CR44] Lhermitte-Vallarino N, Barbuto M, Junker K, Boistel R, Ineich I, Wanji S, Bain O (2009) Rhabdias rhampholeonis n. sp. and Rhabdias mariauxi n. sp.(Nematoda, Rhabdiasoidea), first lung worms from leaf chameleons: Description, molecular evidence and notes on biology. Parasitol Int 58(4):375–383 19646549 10.1016/j.parint.2009.07.009

[CR46] Long PL (1982) The biology of the coccidia. University Park Press, Baltimore, USA, 1982, pp. 502–508

[CR47] Maia JP, Crottini A, Harris DJ (2014) Microscopic and molecular characterization of *Hepatozoon domerguei* (Apicomplexa) and *Foleyella furcata* (Nematoda) in wild endemic reptiles from Madagascar. Parasite 21:47. 10.1051/parasite/2014046 25224723 10.1051/parasite/2014046 PMC4165108

[CR48] Marenzoni ML, Zicavo A, Veronesi F et al (2015) Microbiological and parasitological investigation on chelonians reared in Italian facilities. Vet Ital 51(3):173–178. 10.12834/VetIt.7.21.3 26344661 10.12834/VetIt.7.21.3

[CR49] Marshall BM, Strine C, Hughes AC (2020) Thousands of reptile species threatened by under-regulated global trade. Nat Commun 11:Article 1. 10.1038/s41467-020-18523-4 10.1038/s41467-020-18523-4 PMC7525537 32994397

[CR50] McAllister CT (2012) Two new species of *Isospora* Schneider, 1881 (Apicomplexa: Eimeriidae) from the flap-necked chameleon *Chamaeleo dilepis* (Sauria: Chamaeleonidae) in the Republic of Namibia. Syst Parasitol 83(1):15–20. 10.1007/s11230-012-9361-z 22890376 10.1007/s11230-012-9361-z

[CR51] Mendoza-Roldan JA, Lia RP, Tarallo VD et al (2022) *Raillietiella hemidactyli* (Pentastomida: Raillietiellidae) in *Tarentola mauritanica* geckoes: A new zoonotic parasite for Europe. Acta Trop 228:106316. 10.1016/j.actatropica.2022.106316 35081361 10.1016/j.actatropica.2022.106316

[CR52] Mendoza-Roldan JA, Modry D, Otranto D (2020) Zoonotic parasites of reptiles: A crawling threat. Trends Parasitol 36(8):677–687. 10.1016/j.pt.2020.04.014 32448703 10.1016/j.pt.2020.04.014 PMC7203055

[CR60] Müller A, Wiedmer S, Kurth M (2019) Risk evaluation of passive transmission of animal parasites by feeding of black soldier fly (*Hermetia illucens*) larvae and prepupae. J Food Prot 82(6):948–954 31099595 10.4315/0362-028X.JFP-18-484

[CR56] Modrý D, Daszak P, Volf J et al (2001) Five new species of coccidia (Apicomplexa: Eimeriidae) from Madagascan chameleons (Sauria: Chamaeleonidae). Syst Parasitol 48(2):117–123. 10.1023/A:1006476325181 11252274 10.1023/a:1006476325181

[CR54] Modrý D, Koudela B, Volf J (1997) Four new species of Isospora Schneider, 1881 (Apicomplexa: Eimeriidae) from reptiles from the islands of Seychelles. Syst Parasitol 37(1):73–78

[CR55] Modrý D, Šlapeta JR, Koudela B (2000) Six new species of coccidia (Apicomplexa: Eimeriidae) from East African chameleons (Sauria: Chamaeleonidae). J Parasitol 86(2):373–379. 10.1645/0022-3395(2000)086[0373:SNSOCA]2.0.CO;2 10780560 10.1645/0022-3395(2000)086[0373:SNSOCA]2.0.CO;2

[CR57] Modrý D, Sloboda M (2004) Control of coccidiosis in chameleons using toltrazuril—results of an experimental trial. In: Seybold J, Mutschmann F, eds. Proceedings of the 7th International Symposium on Pathology and Medicine in Reptiles and Amphibians. pp. 93–99

[CR53] Modrý, Koudela DB (1995) Description of *Isospora jaracimrmani* sp. n. (Apicomplexa: Eimeriidae) from the Yemen chameleon *Chamaeleo calyptratus* (Sauria: Chamaeleonidae). Folia Parasitol 42(4):313–316

[CR58] Morsy K, Ramadan N, Hashimi S, Ali M, Bashtar AR (2012) First description of the adult stages of *Postorchigenes* sp. (Trematoda: Lecithodendriidae) and *Malagashitrema* sp. (Trematoda: Homalometridae) infecting the common chameleon *Chamaeleo chamaeleon* (Reptilia: Chamaeleonidae) in Egypt. Life Sci J 9:400–405

[CR59] Mutschmann F (2006) Parasitosen der Reptilien. In: Schneider T (ed) Veterinärmedizinische Parasitologie. Parey, Berlin, pp 739–769

[CR61] Nash MJ, McDermott EA, McGrew AK, Muñoz J, Willems D (2023) A unique disease presentation associated with a mesomycetozoean-like organism in the jeweled chameleon (*Furcifer campani*). J Herpetol Med Surg 33(2):109–115. 10.5818/JHMS-D-22-00033

[CR62] Okulewicz A, Kaźmierczak M, Hildebrand J, Adamczyk M (2015) Endoparasites of lizards (Lacertilia) from captive breeding and trade networks. Helminthologia 52(1):34–40. 10.1515/helmin-2015-0008

[CR63] Palmisano JN, Bockoven C, McPherson SM et al (2022) Infection experiments indicate that common Florida anurans and lizards may serve as intermediate hosts for the invasive pentastome parasite *Raillietiella orientalis*. J Herpetol 56(3):355–361. 10.1670/21-061

[CR64] Pantchev N (2009) Vorkommenshäufigkeit von Endoparasiten bei Reptilien (Schildkröten, Echsen, Schlangen) in Menschenobhut aus der Sicht eines Untersuchungslabors. Tierärztl Umsch 64(4 Suppl):37–43

[CR65] Pantchev N (2011) Parasitenbefall bei Chamäleons in Menschenobhut: Ist das ein Problem? Proc 35. Arbeitstagung der Arbeitsgemeinschaft Amphibien- und Reptilienkrankheiten der DGHT; Bonn, Germany

[CR66] Pantchev N (2013) Parasitosen bei Reptilien. In: Beck W, Panchev N (eds) Praktische Parasitologie bei Heimtieren: Kleinsäuger-Vögel-Reptilien, 2nd edn. Schlütersche, Hannover, pp 283–339

[CR67] Pantchev N, Tappe D (2011) Pentastomiasis and other parasitic zoonoses from reptiles and amphibians. Berl Münch Tierärztl Wochenschr 124(11–12):528–535 22191176

[CR68] Paré JA (2008) An overview of pentastomiasis in reptiles and other vertebrates. J Exot Pet Med 17(4):285–294. 10.1053/j.jepm.2008.07.005

[CR69] Pasmans F, Blahak S, Martel A, Pantchev N (2008) Introducing reptiles into a captive collection: The role of the veterinarian. Vet J 175(1):53–68. 10.1016/j.tvjl.2006.12.009 17346998 10.1016/j.tvjl.2006.12.009

[CR70] Pellett S, Cope I, Fiddes M (2014) Case study: *Foleyella furcata* from a wild-caught graceful chameleon. Companion Anim 19(4):218–221. 10.12968/coan.2014.19.4.218

[CR71] Pike C, Hsieh S, Baling M (2023) Monitoring infection load of oxyurid (nematoda) and *Isospora* (coccidia) in captive inland bearded dragons (*Pogona vitticeps*). Perspect Anim Health Welf 2:77–90. 10.34074/piahw.002106

[CR72] Quiroz-Castañeda RE, Dantán-González E (2015) Control of avian coccidiosis: Future and present natural alternatives. Biomed Res Int 2015:430610. 10.1155/2015/430610 25785269 10.1155/2015/430610 PMC4346696

[CR73] Rataj A, Lindtner-Knific R, Vlahović K, Mavri U, Dovc A (2011) Parasites in pet reptiles. Acta Vet Scand 53:33. 10.1186/1751-0147-53-33 21624124 10.1186/1751-0147-53-33 PMC3118381

[CR74] Reitl K, Ebmer D, Kübber-Heiss A et al (2021) *Hexametra angusticaecoides* (Nematoda: Ascarididae) infection in a panther chameleon (*Furcifer pardalis*): A case report. Wien Tierärztl Monatsschr 11

[CR75] Rinaldi L, Mihalca AD, Cirillo R et al (2012) FLOTAC can detect parasitic and pseudoparasitic elements in reptiles. Exp Parasitol 130(3):282–284. 10.1016/j.exppara.2012.01.011 22306152 10.1016/j.exppara.2012.01.011

[CR76] Rom B, Kornas S, Basiaga M (2018) Endoparasites of pet reptiles based on coproscopic methods. Ann Parasitol 64(2). 10.17420/ap6402.142 10.17420/ap6402.142 29983023

[CR77] Sapion-Miranda P, Ebmer D, Kniha E et al (2025) First identification of a patent pentastomid pulmonary (*Raillietiella orientalis*) infection in a captive Meller’s chameleon (*Trioceros melleri*) in Germany. Int J Parasitol Parasites Wildl 26:101045 40463863 10.1016/j.ijppaw.2025.101045 PMC12130985

[CR78] Satour NS, Dewir AW (2018) Some internal parasites of reptiles in Alexandria Province, Egypt. Egypt Vet Med Soc Parasitol J 14(1):98–114. 10.21608/evmspj.2018.33974

[CR79] Schneller P, Pantchev N (2008) Parasitologie bei Schlangen, Echsen und Schildkröten: Ein Handbuch für die Reptilienhaltung. Edition Chimaira Frankfurt am Main

[CR80] Sergent ME (1902) Sur une coccidie nouvelle parasite du cameleon vulgaire. Comptes Rendus Soc Biol 54:1260–1261

[CR81] Sloboda M, Modrý D (2006) New species of *Choleoeimeria* (Apicomplexa: Eimeriidae) from the veiled chameleon, *Chamaeleo calyptratus* (Sauria: Chamaeleonidae), with taxonomic revision of eimerian coccidia from chameleons. Folia Parasitol 53:91–97. 10.14411/fp.2006.012 16898122

[CR82] Stacy BA (2021) Reptile necropsy techniques. In: Jacobson ER, Garner MM (eds) Infectious diseases and pathology of reptiles: Color atlas and text, 2nd edn. CRC Press, Boca Raton, pp 331–374

[CR83] Stets O (2019) Parasites of panther chameleons (*Furcifer pardalis*) grown in captivity and brought from the wild. J Vet Med Biotechnol Biosaf 5:15–17. 10.36016/JVMBBS-2019-5-4-4

[CR84] Széll Z, Sréter T, Varga I (2001) Ivermectin toxicosis in a chameleon (*Chamaeleo senegalensis*) infected with *Foleyella furcata*. J Zoo Wildl Med 32(1):115–117. 10.1638/1042-7260(2001)032[0115:ITIACC]2.0.CO;2 12790406 10.1638/1042-7260(2001)032[0115:ITIACC]2.0.CO;2

[CR85] Tappe D, Sulyok M, Riu T, ´ozsa R, ´o LB, Schoen I, Muntau CB, Babocsay G, Hardi R (2016) Co-infections in visceral pentastomiasis. Democratic republic of the congo. Emerg Infect Dis 22:1333 27434739 10.3201/eid2208.151895 PMC4982189

[CR88] The IUCN Red List of Threatened Species (2019) IUCN Red List of Threatened Species. https://www.iucnredlist.org/en. Accessed 01 Mar 2023

[CR89] Toland E, Bando M, Hamers M, Cadenas V, Laidlaw R, Martínez-Silvestre A, van der Wielen P (2020) Turning negatives into positives for pet trading and keeping: A review of positive lists. Animals 10(12):2371. 10.3390/ani10122371 33322002 10.3390/ani10122371 PMC7763047

[CR90] Walden M, Mitchell MA (2021) Pathogenesis of *Isospora amphiboluri* in bearded dragons (*Pogona vitticeps*). Animals 11(2):438. 10.3390/ani11020438 33567642 10.3390/ani11020438 PMC7914846

[CR91] Wellehan JFX, Walden HDS (2019) Parasitology (including hemoparasites). In: Divers SJ, Stahl SJ (eds) Mader’s Reptile and Amphibian Medicine and Surgery. Elsevier Health Sciences, St. Louis, pp 281–300.e3

[CR92] Wenyon CM (1920) Observations on the intestinal protozoa of three Egyptian lizards, with a note on a cell-invading fungus. Parasitology 12(4):350–365. 10.1017/S0031182000014347

[CR93] Wilson SC, Carpenter JW (1996) Endoparasitic diseases of reptiles. Semin Avian Exot Pet Med 5(2):64–74. 10.1016/S1055-937X(96)80019-3

[CR94] Wolf D, Vrhovec MG, Failing K, Rossier C, Hermosilla C, Pantchev N (2014) Diagnosis of gastrointestinal parasites in reptiles: Comparison of two coprological methods. Acta Vet Scand 56:44. 10.1186/s13028-014-0044-4 25299119 10.1186/s13028-014-0044-4 PMC4198911

[CR95] Zwart P, Teunis SFM, Cornelissen JMM (1984) Monocercomoniasis in reptiles. J Zoo Anim Med 15(3):129–134

